# Effects of Fucoidan Isolated From *Laminaria japonica* on Immune Response and Gut Microbiota in Cyclophosphamide-Treated Mice

**DOI:** 10.3389/fimmu.2022.916618

**Published:** 2022-05-19

**Authors:** Yunping Tang, Qiuyan Pu, Qiaoling Zhao, Yafeng Zhou, Xiaoxia Jiang, Tao Han

**Affiliations:** ^1^Zhejiang Provincial Engineering Technology Research Center of Marine Biomedical Products, School of Food and Pharmacy, Zhejiang Ocean University, Zhoushan, China; ^2^Zhoushan Institute for Food and Drug Control, Zhoushan, China; ^3^Department of Aquaculture, Zhejiang Ocean University, Zhoushan, China

**Keywords:** fucoidan, cyclophosphamide, immunosuppression, immunomodulatory, gut microbiota

## Abstract

The effects of *Laminaria japonica* fucoidan (LF) on immune regulation and intestinal microflora in cyclophosphamide (CTX)-treated mice were investigated in this work. Results indicated that LF significantly enhanced the spleen and thymus indices, promoted spleen lymphocyte and peritoneal macrophages proliferation, and increased the immune-related cytokines production in serum. Moreover, LF could regulate intestinal flora composition, increasing the abundance of *Lactobacillaceae* and *Alistipes*, and inhibiting *Erysipelotrichia*, *Turicibacter*, *Romboutsia*, *Peptostreptococcaceae*, and *Faecalibaculum*. These results were positively correlated with immune characteristics. Overall, LF could be useful as a new potential strategy to mitigate CTX immunosuppression and intestinal microbiota disorders.

## Introduction

The immune system comprises immune organs, cells, and immune-reactive substances, which play vital roles in the host’s defense against foreign pathogens and microorganisms (1–3). The interaction between the natural and specific immune systems is necessary to maintain host health, and therefore, infections and various diseases will occur when the immune system is dysfunctional or impaired (4). Cyclophosphamide (CTX) is a potent immunosuppressant widely used in treating several immune diseases and malignant tumors (5, 6). However, numerous studies indicated that CTX could disrupt the DNA structure and immune cells and reduce normal lymphocytes and macrophages, thereby inhibiting cellular and humoral immune responses (7, 8). On the other hand, long-term or high doses usage of CTX (80 or 100 mg/kg body weight (BW)) can cause intestinal injury and intestinal microbiota disruption (9–11). Therefore, it is essential to discover effective immunomodulators to reduce the CTX side effects.

Recently, numerous natural polysaccharides have attracted more attention due to their various physiological activities without side effects (12–15). Polysaccharides have been developed as novel immunomodulators, enhancing host immunity and preserving intestinal health (16–18). It has been described that sulfated yam polysaccharide possesses stronger immunomodulatory activity to modulate gut microbiota structure in CTX-treated mice (11). Mulberry leaf polysaccharide has immunomodulatory activity by restoring the injured intestinal barrier and gut microbiota composition in CTX-treated mice (16). At the same time, one report indicated that *Millettia Speciosa Champ* polysaccharide modulates gut health and ameliorates immunosuppression and intestinal injury in CTX-treated mice (17). Considering all these studies, polysaccharides have the potential to be used as an effective immunomodulator to enhance immune function and regulate intestinal flora.

Due to the exploitation and utilization of marine resources, marine natural products have become a research hotspot (19, 20). Fucoidan, a sulfated polysaccharide, has numerous biological activities, including antitumor, antioxidant, anti-inflammatory, immunoregulatory, among others (21–24). *Kjellmaniella crassifolia* or *Undaria pinnatifida* fucoidan could stimulate RAW264.7 cell proliferation *in vitro* and induce a significant immune enhancement *in vivo* (22). Hwang et al. (23) indicated that low-molecular-weight fucoidan from *Laminaria japonica* could enhance the innate and adaptive immune responses and protects against *Mycoplasma pneumoniae* antigen stimulation. Sun et al. (24) indicated that low-molecular-weight fucoidan from *Laminaria japonica* could play an antiviral role by improving the quality of immune organs, improving immune cell phagocytosis and humoral immunity. Nevertheless, there is still a lack of systematic studies regarding the link between the immune regulation function of *Laminaria japonica* fucoidan and intestinal flora.

In our previous studies, we discovered that *Laminaria japonica* fucoidan (LF) could effectively ameliorate CTX-induced liver and kidney injury (25). Herein, the immunomodulatory function of LF was investigated using immunosuppressed mice. In addition, the link between immunomodulatory function and gut microbiota was also evaluated. This systematic research could provide insights into the link between immune regulation function and intestinal flora, and also provided a better understanding of the LF applicability.

## Materials and Methods

### Materials and Reagents

LF, with an average molecular weight of 250 kDa, is composed of mannose, rhamnose, galactose, xylose, and fucose in a molar ratio of 2.04: 0.58: 1.04: 3.91: 12.43, respectively (25). CTX was supplied by Hengrui Medicine (Lianyungang, China). Concanavalin A (ConA) and lipopolysaccharide (LPS) were purchased from Sigma-Aldrich (Sigma-Aldrich, USA). Tumor necrosis factor (TNF)-α, interleukin (IL)-6, and IL-1β ELISA kits were purchased from Boster (Wuhan, China). Immunoglobulin G (IgG) ELISA kit was purchased from Solarbio (Beijing, China).

### Animals and Design

Male ICR mice (18-22 g, 6-8 weeks) were purchased from the Zhejiang Academy of Medical Sciences. All mice were maintained in a breeding environment (23 ± 2°C, 60% ± 5 humidity) on a 12 h light/12 h dark cycle. After 7 days of adaptive feeding, mice were randomly divided into 4 groups (*n* = 8 per group) as follows: control (only treated with a saline solution), model (mice treated with 80 mg/kg BW CTX), 20 LF (mice treated with 20 mg/kg BW LF), and 40 LF (mice treated with 40 mg/kg BW LF). The control group received gavages with saline solution for 19 days (once daily), while the remaining three groups were firstly injected with CTX (80 mg/kg BW) for 5 days (once daily) and then saline solution or 20 and 40 mg/kg BW of LF for another 14 consecutive days (once daily) were received by gavage.

### Spleen and Thymus Indices Determination

The spleen and thymus indices were calculated as follows:


spleen or thymus indices (mg/g)=spleen or thymus weight (mg)/body weight (g)

### Splenic Lymphocyte Proliferation Assay

Splenic lymphocyte was prepared according to Han and collaborators (26). Briefly, the mouse spleen was grinding, and splenic lymphocytes were collected. Next, erythrocyte lysis buffer was added to lyse the red blood cells, and the supernatant was collected by centrifugation (at 1500 × g, 5 min). After removing adherent cells, the cells were incubated in a 6-well plate for 6 h, and the suspended cells were collected as splenic lymphocytes. Then, the cells (1 × 10^6^ cells/mL) were seeded in 96-well plates (final volume of 100 μL). Then, ten microliters of ConA (5 μg/mL) or LPS (1 μg/mL) were added and incubated for 72 h at 37°C with 5% CO_2_. Next, the MTT solution (10 µL, 5 mg/mL) was added, and the absorbance at 490 nm was detected on a microplate reader (Molecular Devices, CA, USA).

### Macrophages Proliferation Assay

The mice were intraperitoneally injected with 5 mL sterile saline solution, and the abdomen was gently pressed for 2 min. After, the abdominal wall was cut open with sterilized scissors, and the abdominal fluid was sucked into a centrifuge tube with a sterile straw. The cell suspension was centrifuged (2000 rpm, 10 min) and resuspended with DMEM medium. The supernatant was discarded to obtain purified macrophages after incubation at 37°C with 5% CO_2_ for 4 h. After this, the cells (1 × 10^4^ cells/mL) were seeded in a 96-well plate (final volume of 200 μL). After 24 h at 37°C with 5% CO_2_, an MTT solution (10 µL, 5 mg/mL) was added, and the absorbance at 490 nm was detected on a microplate reader.

### Phagocytic Activity of Peritoneal Macrophages

The macrophages (1 × 10^4^ cells/mL) were seeded in a 96-well plate (final volume of 200 μL). After 36 h, the medium was discarded, and a neutral red solution (0.1%) was added. After 3 h incubation, the excess neutral red was removed by washing twice with 0.1 M PBS, and lysis solutions (200 μL) were added and shaken for 10 min at 25°C. The absorbance at 540 nm was detected on a microplate reader.

### Serum Cytokines and IgG Content Analysis

The blood was collected from eyeball extirpation, and serum was acquired by centrifugation (6000 × g, 3 min, 4°C) of the blood. The serum levels of TNF-α, IL-6, IL-1β, and IgG were determined using Boster’s ELISA kits.

### Histological Analysis

The spleen and thymus cross-sections were fixed (4% paraformaldehyde) and embedded in paraffin (10%). The hematoxylin-eosin (HE) staining was performed as in previous studies (20, 27). Micrographs were taken using a light microscope CX31 (Olympus, Japan).

### Oxidative Stress Index Analysis

The spleen was ground in saline, and the supernatant was collected by centrifugation. Malondialdehyde (MDA), glutathione peroxidase (GSH-Px), superoxide dismutase (SOD), and catalase (CAT) levels in spleen tissues were measured using Jiancheng’s commercial kits (Nanjing, China).

### Fecal Microbiota Analysis

The genomic DNA of mice feces was extracted, and 338F and 806R primers were used to amplify the V3-V4 region of bacterial 16S rRNA as previously described (27, 28). The MiSeq library was constructed by adding the Illumina official connector sequences to the target area of the PCR products (Shanghai Origingene Bio-pharm Technology Co.Ltd., China). Then, the Illumina Novaseq6000 platform (Illumina, San Diego, USA) was applied for paired sequencing, and the paired-end reads were chosen according to the overlapping relationship. Operational taxonomic units (OTUs) clustering analysis and taxonomic analysis were performed to analyze the diversity indices and community structure at different taxonomic levels, respectively.

### Correlation Between Key Microbes and Immunity-Related Parameters

The correlation heatmap analysis was used to calculate the Spearman rank correlation coefficient between the intestinal key microbial species and basic immunity-related parameters (29). In addition, the microorganisms expressing the highest correlation with LF to enhance the CTX-induced immunosuppression were screened out.

### Statistical Analysis

Results are expressed as the mean ± standard deviation (SD). Data analysis was performed using SPSS 24.0 (SPSS Inc., Chicago, IL, USA). *P* < 0.05 was considered statistically significant.

## Results

### Effect of LF on Spleen and Thymus Indices

**Figure 1:**
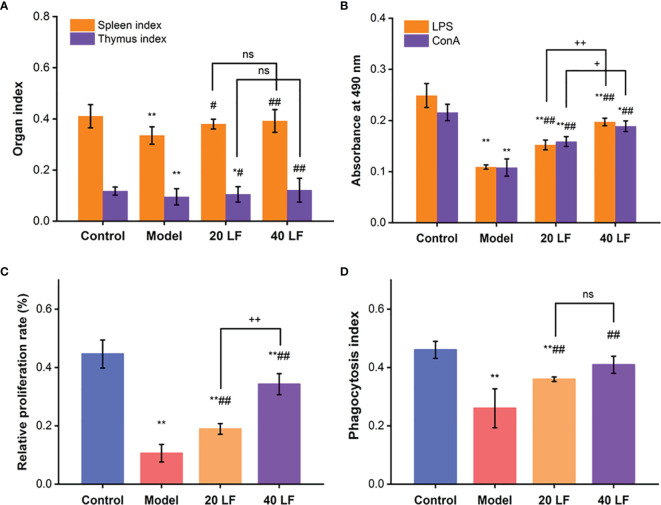
Immune organ indexes of spleen and thymus **(A)**, splenic lymphocytes proliferation **(B)**, peritoneal macrophage proliferation **(C)**, and the phagocytic **(D)** index, (*n* = 8). ^*^P < 0.05, ^**^P < 0.01 *vs* control; ^#^*P* < 0.05, ^##^*P* < 0.01 *vs* model, ^+^*P* < 0.05, ^++^*P* < 0.01 compared between 20 LF and 40 LF, and ns means no significant difference.

In the model group, the spleen and thymus indices were significantly decreased compared to the control group (**Figure 1A**). However, when treated with LF, the spleen and thymus indices showed a significant increase compared to the model (*P* < 0.05 or *P* < 0.01), suggesting that LF treatment could effectively alleviate the CTX-induced atrophy of the spleen and thymus.

### Effect of LF on Splenic Lymphocyte Proliferation

When treated with ConA or LPS, spleen lymphocytes were induced into T and B lymphocytes, respectively (30, 31). Compared to the control, the proliferation of T and B lymphocytes was significantly reduced (*P* < 0.01) in the model, suggesting that CTX could modulate and reduce the cellular immune response (**Figure 1B**). On the other hand, when treated with both LF treatments, the proliferation indices were significantly enhanced compared with the model (*P* < 0.01), suggesting that LF could improve cellular immune response by promoting spleen lymphocytes proliferation. Compared to the model, the macrophages’ relative proliferation rate and the phagocytic index were significantly increased in the 20 LF and 40 LF groups (**Figures 1C, D**, *P* < 0.01), suggesting that LF could promote the macrophages’ proliferation and overcome the immunosuppressed activity induced by CTX.

### Serum Cytokines and IgG Content Analysis

**Figure 2:**
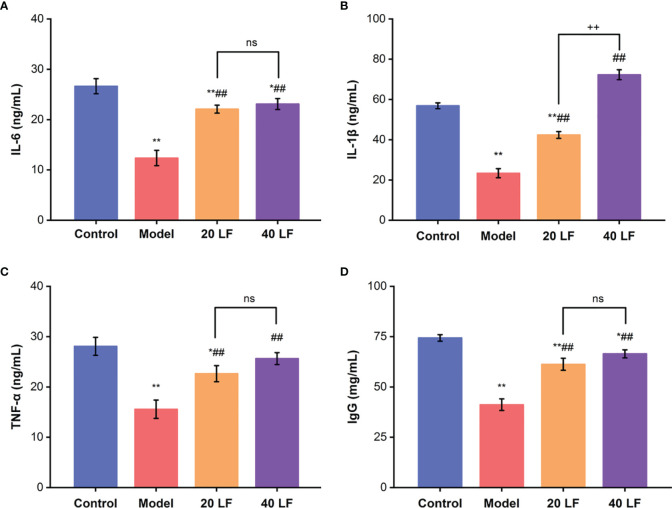
Effects of LF on the serum levels of IL-6 **(A)**, IL-1β **(B)**, TNF-α **(C)**, and IgG **(D)**, (*n* = 8). ^*^P < 0.05, ^**^P < 0.01 *vs* control; ^##^*P* < 0.01 *vs* model, ^++^*P* < 0.01 compared between 20 LF and 40 LF, and ns means no significant difference.

Compared to the control, the IL-6, IL-1β, TNF-α, and IgG levels in the model group were significantly decreased (**Figure 2**, *P* < 0.01), suggesting that CTX inhibited the immune regulation system of mice. However, after treatment with LF, these levels in the 20 LF and 40 LF groups were significantly restored compared to the model (*P* < 0.01), suggesting that LF could improve mice immunosuppression *via* enhancing the cytokines and IgG production.

### Histological Analysis

**Figure 3:**
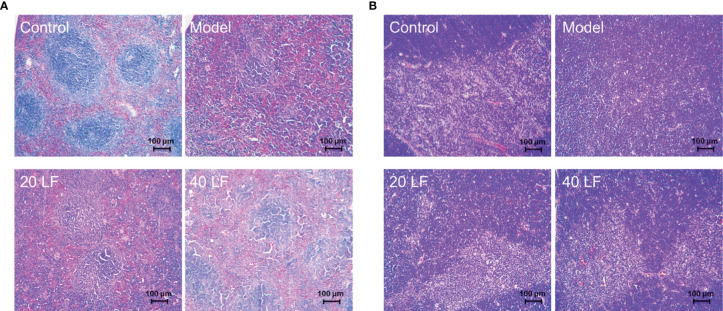
Histomorphology of spleen **(A)** and thymus **(B)** in mice (×100).

To observe the effect of LF on the immune organs, HE staining was applied to detect alterations in the spleen and thymus (**Figure 3A**). The splenic vesicles in the control group remained structurally intact, and a clear demarcation line between the red and white marrow was observed. The splenic cords in the red marrow were connected as a whole, and the blood cells around the exterior of the splenic vesicles were surrounded orderly (**Figure 3A**). However, there were no clearly formed splenic vesicles in the spleen in the model group, and the white and red medullae were partially mixed. The thinning of the lymphatic sheath around the small central artery implies that CTX could damage the spleen immune cells, resulting in spleen atrophy. When treated with 20 mg/kg LF, the general structure of the splenic vesicles could be observed, but the boundary among the red and white medulla remained unclear. Moreover, after treatment with 40 LF, the red and white medulla could be observed, and the marginal area of the white medulla was widened. Overall, the morphology of the spleen in the LF group gradually returned to a similar shape found in the control, suggesting that LF could restore the spleen injury caused by CTX.

Moreover, the cortical and medullary structures of the control were clear and distinct, and evident thymus vesicles could be observed in the medulla (**Figure 3B**). However, when treated with CTX, the structure of the cortex and medulla in the model group was diffused, and few and not evident thymus vesicles were observed in the visual field. On the other hand, there was a clear distinction between the cortex and medulla when treated with LF, mainly in the 40 LF group. Furthermore, the morphology was similar to the control, suggesting that LF could restore the thymus injury caused by CTX.

### Oxidative Stress Index Analysis

**Figure 4:**
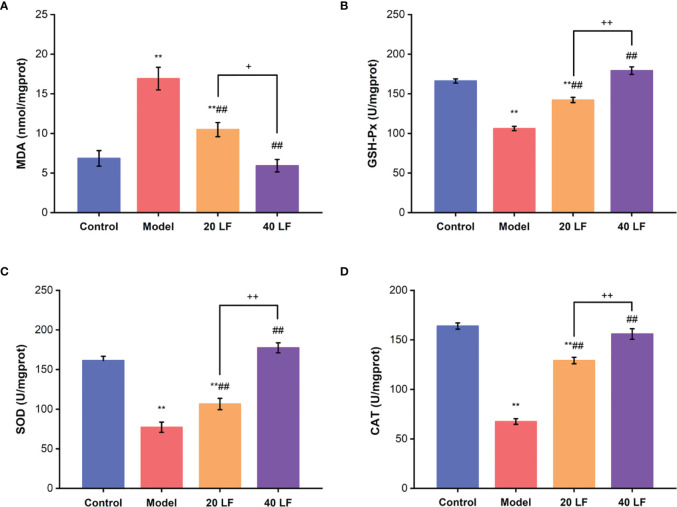
Effects of LF on the MDA levels **(A)**, GSH-Px, SOD, and CAT activities GSH-Px **(B)**, SOD **(C)**, and CAT **(D)** of the spleen (*n* = 8). ^**^P < 0.01 *vs* control; ^##^*P* < 0.01 *vs* model, ^*^P < 0.05, ^++^*P* < 0.01 compared between 20 LF and 40 LF.

The MDA levels and the activities of GSH-Px, SOD, and CAT were also assessed in the spleen. Compared to the control, the MDA contents were significantly increased, and the GSH-Px, SOD, and CAT activities were significantly decreased in the model (**Figure 4**) (*P* < 0.01). After being treated with LF, the MDA contents were significantly reduced (*P* < 0.01), and the GSH-Px, SOD, and CAT activities were significantly increased compared to the model group (*P* < 0.01). These results suggest that LF could improve CTX-induced oxidative stress in the spleen tissues.

### Diversity Analysis of the Intestinal Flora

**Figure 5:**
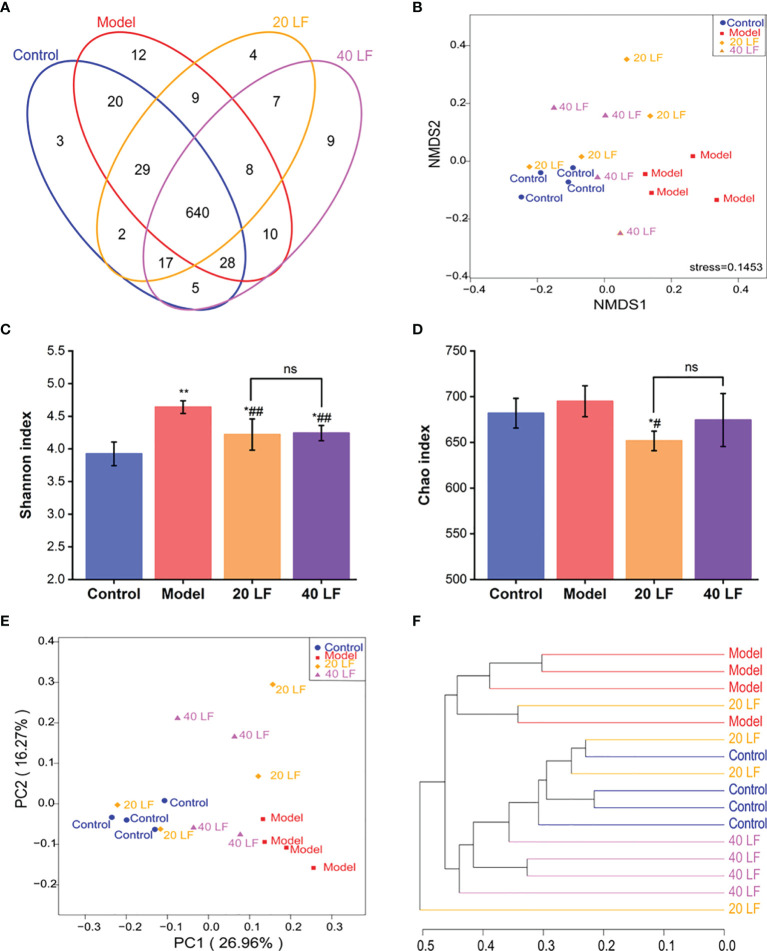
Effects of LF treatment on the gut microbiota composition of the CTX-induced mice (*n* = 4). **(A)** Venn diagrams. **(B)** Non-metric multi-dimensional scaling (NMDS) analysis. **(C)** Shannon indices. **(D)** Chao indices. **(E)** Principal coordinates analysis. **(F)** Cluster tree analysis based on the unweighted UniFrac at the genus level. ^*^P < 0.05, ^**^P < 0.01 *vs* control; ^#^*P* < 0.05, ^##^*P* < 0.01 *vs* model, and ns means no significant difference.

To investigate the LF effect on intestinal microflora composition in mice, a 16S rRNA sequencing was performed. The Venn diagram showed common and unique OTUs between different samples (**Figure 5A**), and 803 OTUs in all samples were identified. Additionally, 640 shared OTUs in the four groups, accounting for 79.70% of the total OTUs, indicating that the bacterial compositions of all groups were similar. Non-metric multi-dimensional scaling (NMDS) analysis can reflect the relationship between microbial communities of different samples. As shown in **Figure 5B**, there are significant differences in the microbial community structure between the control and the model group, while the microorganisms of the 20 LF and 40 LF groups are relatively similar to those of the control group. In addition, the alpha diversity change (Shannon and Chao indices) was shown in **Figures 5C, D**. Except for the Chao index of the 40 LF group, the results of alpha diversity were significantly different from the CTX group after different doses of LF intervention (*P* < 0.05), indicating that LF could reverse the changes in the abundance and diversity of fecal bacteria caused by CTX.

Principal coordinates analysis (PCOA) and cluster tree analysis were performed to analyze the similarity of microbial communities among different groups. The PCOA results suggested that CTX and LF intervention significantly affected the intestinal flora composition (**Figure 5E**). Furthermore, the distance between the 40 LF and control groups was smaller than that between the control and model groups, suggesting that the intervention of LF could cause a shift in gut flora composition. In addition, the cluster analysis results based on bray curtis distance were consistent with the PCOA results (**Figure 5F**), suggesting that CTX and LF could effectively influence the intestinal flora composition. In addition, the intestinal flora of the LF group was more similar to the control.

### Analysis of Community Differences of Intestinal Flora

**Figure 6:**
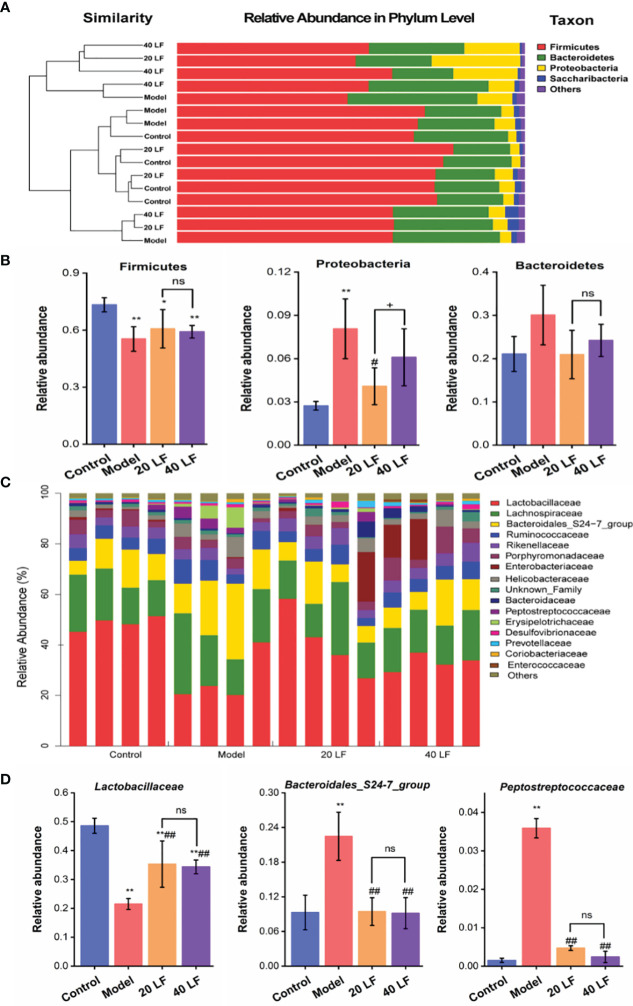
Comparisons of the gut microbiota at phylum and family taxonomic levels (*n* = 4). **(A)** Gut microbiota composition at the phylum level. **(B)** Relative abundance of the gut microbiota at the phylum level. **(C)** Gut microbiota composition at the family level. **(D)** Relative abundance of the gut microbiota at the family level. ^*^P < 0.05, ^**^P < 0.01 *vs* control; ^#^*P* < 0.05, ^##^*P* < 0.01 model, ^+^*P* < 0.05 compared between 20 LF and 40 LF, and ns means no significant difference.

To identify specific taxa associated with LF, we assessed the relative abundance of species at phylum and family levels. The mouse intestinal flora comprises Firmicutes, Bacteroidetes, and Proteobacteria, together accounting for over 90% (**Figure 6A**). The differences in the relative abundance of these three major bacteria were further analyzed (**Figure 6B**). It was observed that CTX treatment significantly decreased the abundance of Firmicutes and significantly increased the abundance of Proteobacteria (*P* < 0.05). After LF treatment, the changes of relative abundance of the three phyla were reversed, but only changes of Proteobacteria abundance in the 20 LF group were significantly different. The gut flora varied significantly at the family level (**Figure 6C**), where the top 10 species with relative abundance were *Lactobacillaceae*, *Lachnospiraceae*, *Bacteroidales*_S24-7_group, *Ruminococcaceae*, *Rikenellaceae*, *Porphyromonadaceae*, *Enterobacteriaceae*, *Helicobacteraceae*, *Bacteroidaceae*, and *Peptostreptococcaceae* (**Table 1**). CTX significantly altered the relative abundance of *Lactobacillaceae*, *Bacteroidales*_S24-7_group, and *Peptostreptococcaceae* compared to the control, and LF treatment at different doses reversed these changes caused by CTX (**Figure 6D**, *P* < 0.01).

**Table 1:** Changes of intestinal microorganisms in the top 10 relative abundance at the family level (*n* = 4).

Microorganisms	Control	Model	20 LF	40 LF
*Lactobacillaceae*	0.486 ± 0.026	0.215 ± 0.019 ^**^	0.353 ± 0.080 ^**^^##^	0.344 ± 0.024 ^**^^##^
*Lachnospiraceae*	0.164 ± 0.036	0.285 ± 0.049 ^**^	0.179 ± 0.074	0.175 ± 0.019
*Bacteroidales_S24-7_group*	0.093 ± 0.030	0.225 ± 0.042 ^*^	0.094 ± 0.024 ^##^	0.092 ± 0.027 ^##^
*Ruminococcaceae*	0.052 ± 0.006	0.083 ± 0.012 ^**^	0.050 ± 0.020	0.058 ± 0.008
*Porphyromonadaceae*	0.050 ± 0.018	0.029 ± 0.013	0.035 ± 0.007	0.072 ± 0.033 ^#^
*Rikenellaceae*	0.044 ± 0.009	0.029 ± 0.008	0.050 ± 0.014	0.054 ± 0.014
*Helicobacteraceae*	0.017 ± 0.007	0.053 ± 0.026 ^*^	0.031 ± 0.019	0.035 ± 0.022
*Bacteroidaceae*	0.008 ± 0.002	0.004 ± 0.003	0.021 ± 0.027	0.017 ± 0.016
*Enterobacteriaceae*	0.007 ± 0.002	0.001 ± 0.0006 ^*^	0.053 ± 0.007	0.043 ± 0.074
*Peptostreptococcaceae*	0.0015 ± 0.0008	0.0359 ± 0.0025 ^**^	0.0047 ± 0.0006 ^##^	0.0024 ± 0.0015 ^##^

^*^P < 0.05, ^**^P < 0.01 vs the Control, ^#^P < 0.05, ^##^P < 0.01 vs model.

### LEfSe Analysis of Intestinal Flora in Mice

**Figure 7:**
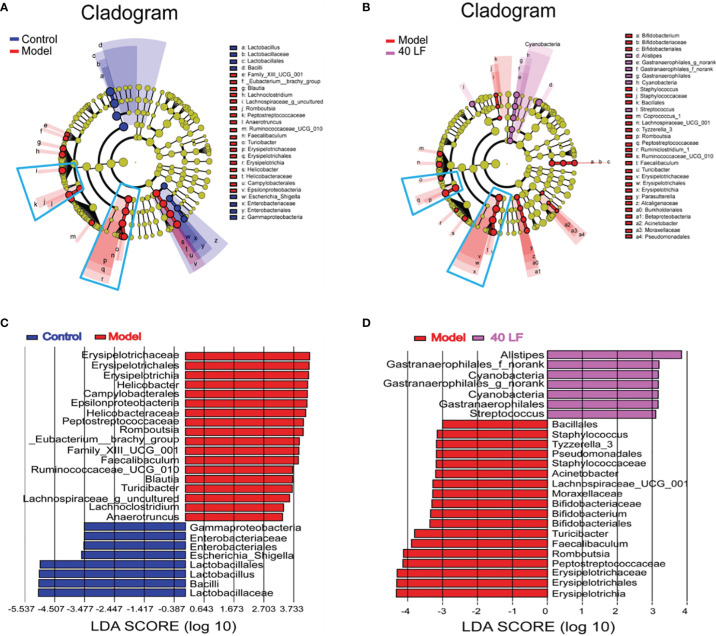
The LEfSe and Spearman correlation analyses of the fecal microbes of the different mice groups (*n* = 4). **(A)** LEfSe analysis showing the key differential microbes of the CTX-induced mice. **(B)** LEfSe analysis showing the key differential microbes of the CTX-induced immunosuppressed mice subjected to the 40 LF intervention. **(C)** The LDA score between the control and the model groups. **(D)** The LDA score between the model and the 40-LF treated groups.

LEfSe was performed to identify taxa with significant differences in abundance, while linear discriminant analysis (LDA) was performed to estimate the influence of the abundance of each component on the differential effect. From the Lefse results (**Figure 7**), it was observed that the CTX treatment mainly suppressed the relative abundance of *Lactobacillales*, *Bacilli*, *Enterobacteriales*, and *Gammaproteobacteria*, and promoted the relative abundance of *Family*_XIII_UCG_001, *Eubacterium*_brachy_group, *Blautia*, *Lachnoclostridium*, *Romboutsia*, *Peptostreptococcaceae*, *Anaerotruncus*, *Ruminococcaceae*_UCG_010, *Faecalibaculum*, *Turicibacter*, *Erysipelotrichia*, *Helicobacteraceae*, *Campylobacterales*, and *Epsilonproteobacteria*. Compared to the model, 40 LF treatment significantly suppressed the relative abundance of the CTX-stimulated microorganisms, especially the relative abundance of *Erysipelotrichia*-related groups, and promoted the relative abundance of *Alistipes*, *Gastranaerophilales*, *Cyanobacteria*, and *Streptococcus*.

### Correlation Between Key Microbes and Host Parameters

**Figure 8:**
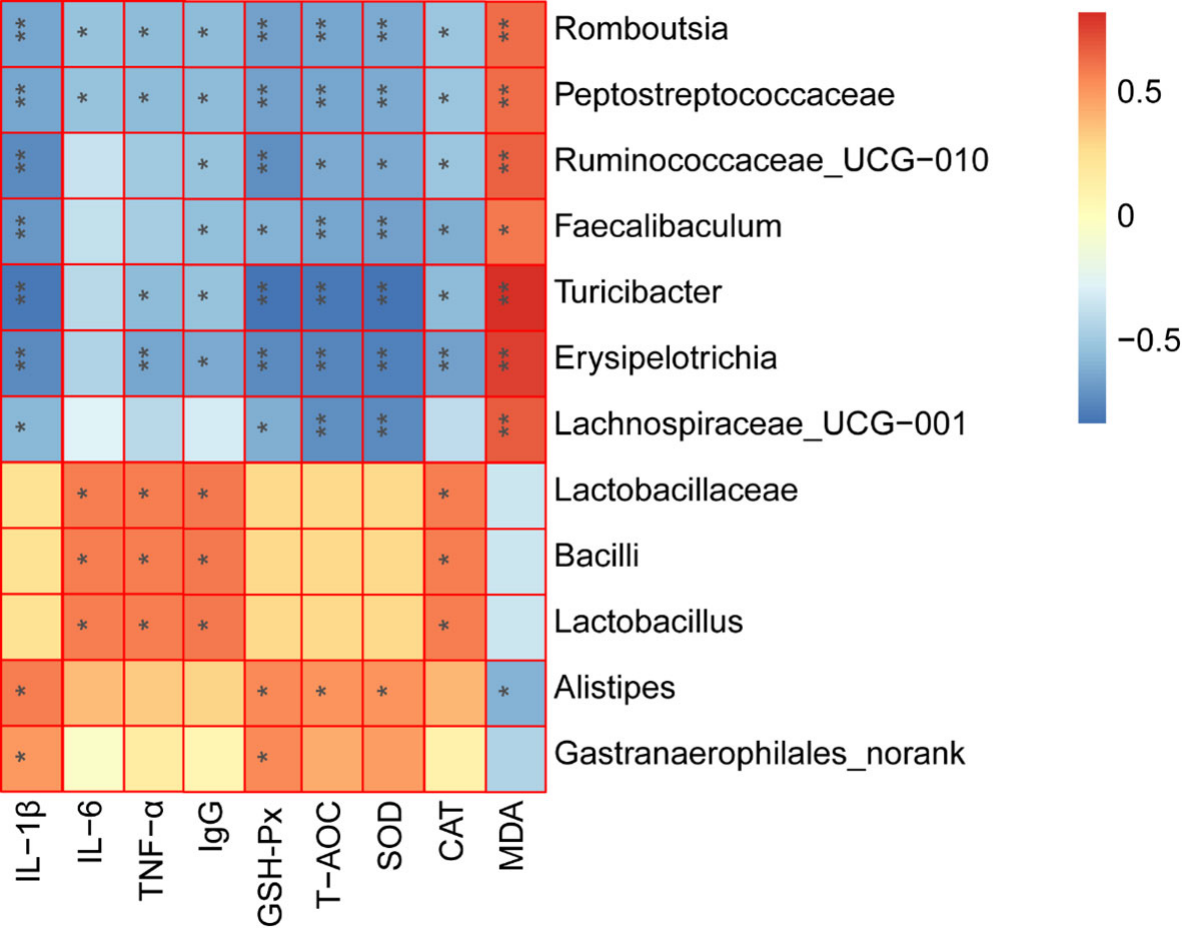
Spearman correlation analysis between the key microbial communities and immune-related biochemistry parameters. *P < 0.05, **P < 0.01.

The correlation heatmap analysis was used to calculate the Spearman rank correlation coefficient between the intestinal key microbial species and host parameters. In addition, the key microorganisms expressing the highest correlation with LF to enhance the CTX-induced immunosuppression were screened out. As depicted in **Figure 8**, 5 types of microbes (*Romboutsia*, *Peptostreptococcaceae*, *Faecalibaculum*, *Turicibacter*, and *Erysipelotrichia*) presented a significant negative correlation with the CAT, SOD, and GSH-Px activities, pro-inflammatory cytokine levels (IL-1β and TNF-α), while also being positively correlated with the MDA content (*P* < 0.05). These bacteria were the key species of intestinal microbiota in the model group and were also inhibited by LF. In contrast, 3 types of key microorganisms (*Lactobacillaceae*, *Bacilli*, and *Lactobacillus*) presented a positively correlated with pro-inflammatory cytokine levels (IL-6 and TNF-α), IgG, CAT activity. Moreover, the LF group was also enriched with *Alistipe*s, presenting a significant negative correlation with MDA levels and positively correlated with SOD and GSH-Px activities.

## Discussion

CTX is an effective immunosuppressant, widely applied as a vital drug in cancer treatment and both blood and bone marrow transplants (5). However, CTX treatment can induce immunosuppression and intestinal flora dysfunction (32, 33). Polysaccharides could be used to alleviate the CTX-induced immunosuppression and intestinal dysbacteriosis (16, 17). For example, Mulberry leaf-derived polysaccharide has the potential to modulate the immune response and gut microbiota composition in immunosuppressed mice (16). Millettia Speciosa Champ roots polysaccharides could modulate gut health and ameliorate CTX-induced intestinal injury and immunosuppression (17). In this work, immunosuppression was induced in mice to investigate the possible positive effects of LF treatment on such immunosuppression, as well as the intestinal flora dysfunction caused by CTX.

The ability of the spleen to induce lymphocytic proliferation is an important index of the body’s immune response (26). As another important immune cell, macrophages exist in various cellular tissues and, together with neutrophils, constitute the first line of the body’s immune defense, playing vital roles in non-specific and specific immunity (30). Macrophages are phagocytes derived from monocytes and play key roles in natural and specific immune systems (31). Activated macrophages can actively phagocytose and remove foreign antigens or directly kill pathogenic microorganisms. In this study, CTX significantly decreased the mice’s T and B lymphocyte proliferation, suggesting an impaired cellular immune response, consistent with previous studies (26, 34). However, LF (20 mg/kg or 40 mg/kg) treatment could significantly promote the T and B lymphocytes proliferation. Moreover, the proliferative capacity and phagocytosis indices of peritoneal macrophages were also significantly increased after LF treatment. These results showed that LF treatment ameliorated the CTX injury to immune cells and enhanced the immune response of mice.

Cytokines are soluble extracellular polypeptides or glycoproteins indispensable in the immune response (29, 32). Immunoglobulins, secreted by B cells, are an important part of the immune response (29). CTX could decrease the serum levels of cytokines or immunoglobulins, causing a decrease in immunity (29, 31). Our results indicated that the IL-6, IL-1β, TNF-α, and IgG serum levels were decreased in the model group, which was consistent with previous studies (29, 31). However, after treatment with LF, both cytokines (IL-6, IL-1β, TNF-α) and IgG serum levels were significantly increased compared with the model, indicating that LF could promote IL-6, IL-1β, TNF-α, and IgG serum levels to improve immune function, consistent with the previous studies (23). The spleen and thymus are two important immune organs (35). In this work, the spleen and thymus indices were decreased significantly after CTX treatment, and HE staining results also indicated that CTX could induce apoptosis of the spleen and thymus, but LF treatment could reverse these adverse effects, suggesting that LF had a rescue effect. Sun et al. (24) that low-molecular-weight fucoidan from *Laminaria japonica* could increase the thymus and spleen index in the virus-infected mice, and our results were consistent with their studies. Furthermore, previous studies have shown a correlation between immune ecology and oxidative stress, and immune organ damage is closely related to oxidative stress (29, 36). Moreover, the dynamic balance between the oxidative and antioxidant states of the body plays a vital role in safeguarding the organism’s health (30, 37). Polysaccharides have strong antioxidant properties and can ameliorate the CTX-induced low immunity by alleviating oxidative stress damage (30, 38). In this work, the spleen levels of GSH-Px, CAT, and SOD were significantly decreased, and MDA levels were significantly increased in the model group, consistent with previous studies (38). While GSH-Px, CAT, and SOD levels were significantly enhanced after LF treatment, MDA levels were significantly reduced. These results suggested that LF could ameliorate the CTX-induced oxidative stress, consistent with our previous studies (25).

Gut flora plays key roles in shaping the immune system (39). CTX could modulate the intestinal flora composition, while polysaccharides also can modulate intestinal flora composition after CTX treatment, thus regulating the host’s immunity (11, 29). In this work, after CTX treatment, the relative abundances of *Lactobacillaceae*, *Porphyromonadaceae*, *Rikenellaceae*, *Bacteroidaceae*, and *Enterobacteriaceae* were decreased, and the relative abundances of *Bacteroidales_S24-7_group*, *Ruminococcaceae, Lachnospiraceae*, *Helicobacteraceae*, and *Peptostreptococcaceae* were significantly increased. These results are in agreement with the work performed by Ding and collaborators (40). However, LF can reverse the CTX-induced changes in the abundance of bacteria at the family level. A comprehensive analysis of Lefse and community composition found that LF treatment significantly reversed the intestinal flora disturbance caused by CTX by significantly inhibiting the relative abundance of *Erysipelotrichia*, *Peptostreptococcaceae*, *Faecalibaculum*, *Turicibacter*, *Romboutsia*, and *Helicobacteraceae*. The relative abundance of *Erysipelotrichia* related groups, and beneficial strains, including *Lactobacillaceae*, *Alistipes*, *Gastranaerophilales*, *Cyanobacteria*, and *Streptococcus*, increased significantly. Further, the Spearman rank correlation coefficient was used to calculate the association between the intestinal key microbial species and host parameters. The results showed that 5 types of key microorganisms (*Romboutsia*, *Peptostreptococcaceae*, *Faecalibaculum*, *Turicibacter*, and *Erysipelotrichi*) were negatively correlated with immune characteristics, and 4 types of key microorganisms (*Lactobacillaceae*, *Bacilli*, *Lactobacillus*, and *Alistipes*) were positively correlated with immune characteristics.

## Conclusion

In summary, LF enhanced the immune response by enhancing the secretion of cytokines and IgG, alleviating spleen and thymus injury in CTX-treated mice. In addition, LF could also regulate the intestinal flora disorder caused by CTX. These findings indicated that LF has the potential to be used as an immunoregulatory adjuvant or functional food additive to ameliorate CTX-induced immunosuppression.

## Data Availability Statement

The datasets presented in this study can be found in online repositories. The names of the repository/repositories and accession number(s) can be found below: https://www.ncbi.nlm.nih.gov/genbank/, PRJNA830087.

## Ethics Statement

The animal study was reviewed and approved by Animal Ethics Committee of Zhejiang Ocean University.

## Author Contributions

YT and TH conceived the study and designed the project. YT, QP, QZ, YZ, and XJ performed the experiment and analyzed the data. YT drafted the manuscript. YT and TH revised the manuscript and supervised the whole study. All authors contributed to the article and approved the submitted version.

## Funding

This work was financially supported by the Zhoushan Science and Technology Project (No.2022C41004), and the National Natural Science Foundation of China (No. 41806153).

## Conflict of Interest

The authors declare that the research was conducted in the absence of any commercial or financial relationships that could be construed as a potential conflict of interest.

## Publisher’s Note

All claims expressed in this article are solely those of the authors and do not necessarily represent those of their affiliated organizations, or those of the publisher, the editors and the reviewers. Any product that may be evaluated in this article, or claim that may be made by its manufacturer, is not guaranteed or endorsed by the publisher.
